# Antifungal Activity and Potential Mechanism of N-Butylphthalide Alone and in Combination With Fluconazole Against *Candida albicans*

**DOI:** 10.3389/fmicb.2019.01461

**Published:** 2019-07-02

**Authors:** Ying Gong, Weiguo Liu, Xin Huang, Lina Hao, Yiman Li, Shujuan Sun

**Affiliations:** ^1^ Department of Pharmacy, Shandong Provincial Qianfoshan Hospital, Shandong University, Jinan, China; ^2^ School of Pharmaceutical Sciences, Shandong University, Jinan, China; ^3^ Department of Pharmacy, Shandong Provincial Qianfoshan Hospital, The First Hospital Affiliated with Shandong First University, Jinan, China; ^4^ Department of Pharmacy, Qilu Children’s Hospital of Shandong University, Jinan, China

**Keywords:** antifungal activity, *Candida albicans*, fluconazole, n-butylphthalide, potential mechanism

## Abstract

*Candida albicans* is a common opportunistic fungal pathogen that may cause nosocomial fungal infections. The resistance of *Candida albicans* to traditional antifungal drugs has been increasing rapidly in recent years, and it brings a great challenge in clinical treatment. N-butylphthalide is originally extracted from the seed of *Apium graveolens* and is currently used for the treatment of ischemic stroke in the clinic. This study demonstrated that n-butylphthalide exhibited antifungal activity against *Candida albicans* with minimum inhibitory concentrations of 128 μg/ml; moreover, n-butylphthalide combined with fluconazole showed synergistic antifungal effects against resistant *Candida albicans*, resulting in a decrease in the minimum inhibitory concentrations of fluconazole from >512 to 0.25–1 μg/ml. Time-killing curves verified the antifungal activity in dynamic. Besides, n-butylphthalide exhibited anti-biofilm activity against *Candida albicans*, biofilms preformed <12 h with sessile minimum inhibitory concentrations of 128–256 μg/ml and synergism was observed when n-butylphthalide combined with fluconazole against resistant *Candida albicans* biofilms preformed <12 h, resulting in a decrease in the sessile minimum inhibitory concentrations of fluconazole from >1,024 to 0.5–8 μg/ml. Furthermore, *in vitro* antifungal effects of n-butylphthalide were confirmed *in vivo*. N-butylphthalide prolonged survival rate of larvae infected by *Candida albicans*, reduced the fungal burden in larvae and caused less damage to larval tissues. Notably, n-butylphthalide inhibited hyphal growth and induced intracellular reactive oxygen species accumulation and a loss in mitochondrial membrane potential, which was a potential antifungal mechanism. Besides, the synergistic effects between n-butylphthalide and fluconazole potentially relied on the mechanism that n-butylphthalide significantly promoted drug uptake, and suppressed drug efflux *via* down-regulating the drug transporter encoding genes *CDR1* and *CDR2*. These findings demonstrated the antifungal effects and mechanisms of n-butylphthalide against *Candida albicans* for the first time, which might provide broad prospects for the identification of new potential antifungal targets.

## Introduction

Due to the extensive application of broad-spectrum antibiotics, immunosuppressive agents, and medical implant devices, the incidence of fungal infections has increased rapidly in the last few decades (Suleyman and Alangaden, 2016). The leading *Candida* species, *Candida albicans* (*C. albicans*), is the most common fungal pathogen that may cause epidermal and potentially life-threatening invasive infections, especially in immunocompromised patients (Dimopoulos et al., 2007). Fluconazole (FLC), a kind of azoles, is the most frequently used antifungal drug for prevention and treatment of *C. albicans* infections due to the high efficacy and low toxicity. However, drug resistance to antifungals, especially to FLC among *C. albicans* species, increased sharply along with long-term use of it (Whaley et al., 2016). Furthermore, biofilms adhered on the abiotic and biotic surfaces act as the natural barrier to the dispersion of antifungal drugs and are inherently resistant to most antifungal drugs (Bonhomme and d’Enfert, 2013; Desai et al., 2014). Thus, there is an urgent need to develop therapeutic strategies to combat drug resistance of *C. albicans*.

**Figure 1:**
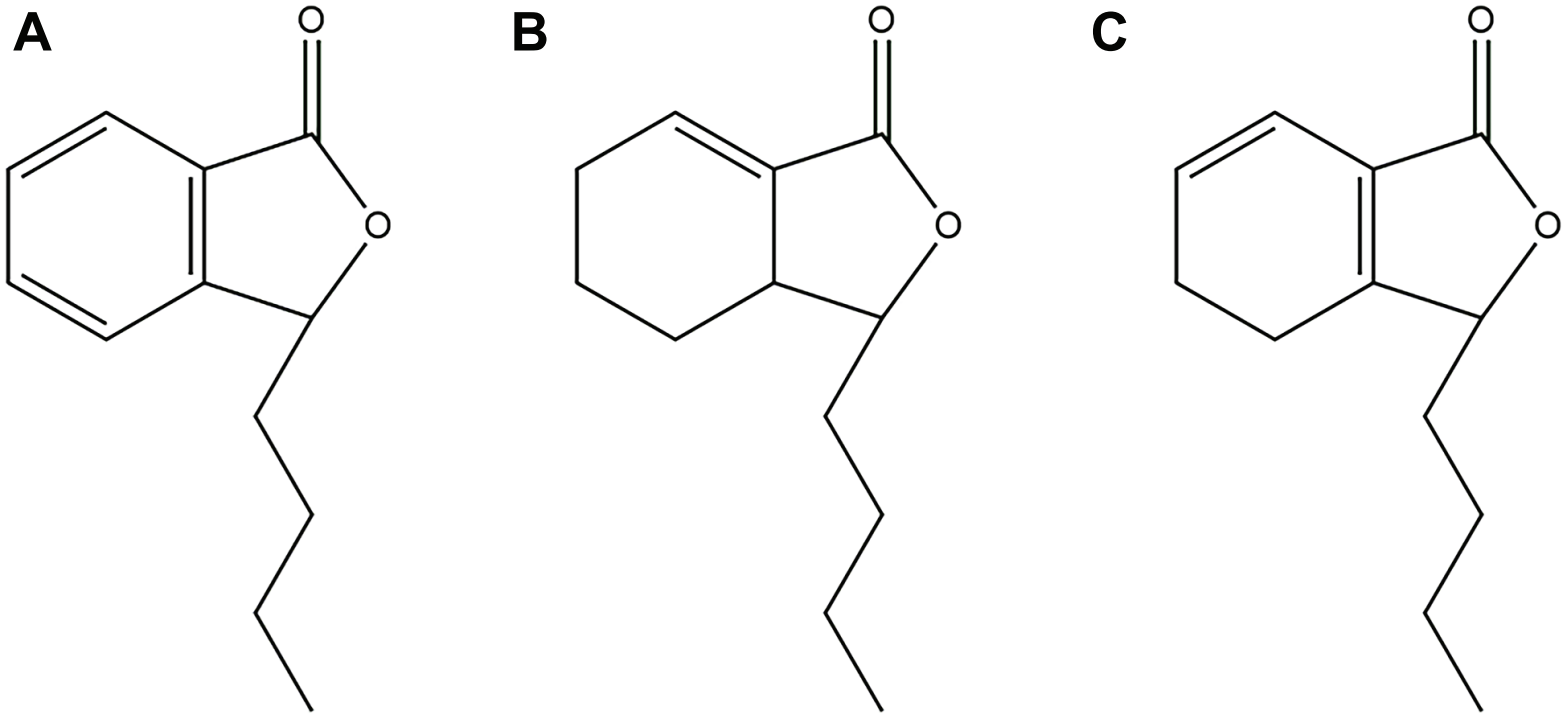
**(A)** Chemical structure of NBP. **(B)** Chemical structure of compound1. **(C)** Chemical structure of compound2.

Natural products, especially extracted from traditional Chinese herbal medicine, provide a huge treasure pool for drug discovery by serving as compounds with metabolic activity in their natural form or synthetic modification (Mishra and Tiwari, 2011). It is worth noting that phytocompounds exhibit prominent potential as antifungal agents or as synergistic agents with FLC, particularly against *Candida* spp. (Lu et al., 2017). For example, Shao et al. confirmed that sodium houttuyfonate revealed relatively strong antifungal potential against *C. albicans* (Huang et al., 2015; Shao et al., 2017; Da et al., 2019). N-butylphthalide (NBP) (Figure 1A) is originally extracted from the seed of *Apium graveolens*, and it is a new drug that has been independently researched and developed in China (Zhao et al., 2014). NBP has a wide range of pharmacological effects on cerebrovascular diseases: resisting cerebral ischemia, improving brain cell energy metabolism, and inhibiting thrombosis. Currently, NBP is widely used in the clinic for the treatment of ischemic stroke because of its low toxicity and good safety (Zhao et al., 2014; Abdoulaye and Guo, 2016). Furthermore, it has been confirmed that compound1 (Figure 1B) and compound2 (Figure 1C) are the structural analogues of NBP and are also extracted from *Apium graveolens* seeds, have antifungal activity against *C. albicas* (Momin et al., 2000; Momin and Nair, 2001). However, at present, there are no reports investigating the antifungal activity of NBP, alone and combined with FLC, against *C. albicans*.

In this study, the *in vitro* antifungal effects of NBP alone and in combinations with FLC against planktonic *C. albicans* and biofilms in different stages (4, 8, 12, and 24 h) were evaluated. The dynamical antifungal effects of NBP were demonstrated by time-killing curves. In addition, *Galleria mellonella (G. mellonella)-C. albicans* infection model was established and the survival rate, fungal burden, and histopathology were used to evaluate the effects of NBP *in vivo.* For the exploration of underlying mechanism, we investigated the effects of NBP on hyphal growth, the levels of intracellular reactive oxygen species (ROS) and mitochondrial membrane potential (Δ*ψ_m_*). Furthermore, to explore the potential synergistic mechanism of NBP combined with FLC, we conducted rhodamine 6G assays to detect the effects of NBP on drug uptake and efflux of resistant strains. We also carried out real-time quantitative PCR assays (RT-PCR) to determine the gene expression levels of *CDR1, CDR2*, and *MDR1*, which encode efflux pump proteins.

## Materials And Methods

### Strains, Culture, and Agents

Six *C. albicans* strains were used in this study, including two FLC-susceptible strains (CA4 and 8) and four FLC-resistant strains (CA10, 16, 103, and 632). CA4, 8, 10, and 16 were collected from the clinical laboratory at Qianfoshan Hospital Affiliated to Shandong University (Jinan, China), and CA103 and 632 were kindly provided by Professor Changzhong Wang (School of integrated traditional and western medicine, Anhui University of traditional Chinese medicine, Hefei, China). Their susceptibilities were determined according to Clinical and Laboratory Standards Institute (CLSI) document M27-A3 (Institute CaLS, 2008a,b). *C. albicans* ATCC 10231, kindly provided by the Institute of Pharmacology, School of Pharmacy, Shandong University (Jinan, China), was used as the quality control strain. CA10 was used as a representative strain for time-killing test, *in vivo* experiment and mechanism exploration. Strains were refreshed from the frozen stocks at −80°C and inoculated at least twice onto sabouraud solid medium for 18 h at 35°C before all experiments. RPMI 1640 (pH 7.0) was used as the liquid medium for diluting drugs and strains.

All drugs (NBP, penicillin sodium and FLC) were purchased from Dalian Meilun Biotech Co. Ltd., China. Stock solution of NBP was dissolved in absolute ethyl alcohol with 0.5% tween80 at a final concentration of 12,800 μg/ml. Stock solutions of penicillin sodium and FLC were prepared in sterile distilled water to a final concentration of 2560 μg/ml. All stock solutions were stored at −20°C until use.

### Determination of Minimum Inhibitory Concentrations of Planktonic Cells

The minimum inhibitory concentrations (MICs) of NBP alone and in combination with FLC against *C. albicans* isolates were determined with a broth microdilution method as described by the CLSI guidelines (Institute CaLS, 2008a,b). The tests were performed in 96-well flat-bottomed microtiter plates. The final concentration of fungal suspension in RPMI 1640 medium was 10^3^CFU/ml, the final concentration of NBP ranged from 4 to 256 μg/ml and the final concentration of FLC ranged from 0.125 to 64 μg/ml. All of the wells were filled with RPMI 1640 to a final volume of 200 μl. A drug-free well served as a growth control, and wells containing RPMI 1640 medium only were set as negative controls. Plates were incubated at 35°C for 24 h. The growth inhibition was determined both by visual reading and by measuring the optical density at 492 nm using a microplate reader. MIC_80_ was defined as the lowest concentration of drug, alone and in combination that inhibited the growth of yeast by 80% compared with the control group (Lewis et al., 2002; Li et al., 2011; Khan and Ahmad, 2012). The *in vitro* interaction of the drug combination was interpreted in terms of the fractional inhibitory concentration index (FICI) (Odds, 2003). The FICI model was expressed as follows: FICI = FIC_FLC_ + FIC_NBP_ = (MIC_80_ of FLC in combination/MIC_80_ of FLC alone) + (MIC_80_ of NBP in combination/MIC_80_ of NBP alone). The interpretation of the FICI was defined as FICI of ≤0.5 for synergy, FICI >4.0 for antagonism and 0.5 < FICI≤4.0 for no interaction.

### Determination of Sessile Minimum Inhibitory Concentrations of *C. albicans* Biofilms

Sessile MICs (SMICs) of NBP, alone and combined with FLC against *C. albicans* (CA4, 10 and 16), were evaluated as described by Ramage and Lopez-Ribot (2005) with moderate modifications. In brief, biofilms were formed by adding 200 μl cell suspension (10^3^CFU/ml) into 96-well flat-bottomed microtiter plates over four time intervals (4, 8, 12, and 24 h) at 35°C. At each time point, each well was washed with 200 μl PBS three times to remove the planktonic and nonadherent cells. Subsequently, drugs of different concentrations were added and the plates were incubated for another 24 h at 35°C. The final concentration of FLC and NBP in wells ranged from 0.125 to 64 μg/ml and from 4 to 256 μg/ml, respectively. A metabolic assay based on the reduction of 2,3–bis (2–methoxy–4–nitro–5–sulfophenyl)–2H–tetrazolium–5–carboxanilide (XTT) was carried out to determine the sMICs. SMIC_80_ referred to the lowest concentrations, where there was an 80% reduction in the XTT-colorimetric readings compared with the drug-free control (Prazynska and Gospodarek, 2014; Zhong et al., 2017). Colorimetric absorbance was measured at 492 nm in a microtiter plate reader. The FICI model was used to illustrate the interaction between NBP and FLC against *C. albicans* biofilms as described above.

### Time-Killing Curve Assay

Groups containing NBP (64, 128, and 256 μg/ml, respectively), FLC (1 μg/ml), NBP/FLC (128 and 1 μg/ml, respectively) and 10^5^CFU/ml of *C. albicans* suspension were then incubated at 35°C with constant shaking (200 rpm). The group with no drug was served as a control growth group. At prearranged time points (0, 6, 12, 24, and 48 h) after incubation, the amount of living cells was then measured by colony counting methods (Li et al., 2008, 2015; Shrestha et al., 2015). For judgment of the interaction between NBP and FLC, synergism was defined as a ≥ 2lg10 decrease in CFU/ml and indifference as a <2lg10decrease in CFU/ml compared to the most active drug, and antagonism as a ≥ 2lg10 increase in CFU/ml compared to the least active drug (Li et al., 2014b).

### Determination of *in vivo* Antifungal Effects by *G. mellonella* Infection Model

Three *in vivo* experiments, survival assay, fungal burden determination and histological study, were carried out. The initial steps of each experiment were identical. *G. mellonella* larvae during the last instar of the larval development were selected to be absent of dark spots and similar in size (approximately 0.25 ± 0.02 g). Each group contained 20 randomly chosen larvae and they were placed in perish dishes at 35°C overnight before experiments. About 10 μl of *C. albicans* suspension (10^8^CFU/ml) was inoculated directly to the last left pro-leg. Before injection, the area was swabbed by ethanol for disinfection. After 2 h injection, where four groups of the larvae were injected *via* the last right pro-leg with 10 μl of sterile PBS, NBP (40 μg/ml), FLC (160 μg/ml), and NBP + FLC (40 +160 μg/ml), all groups of larvae were incubated at 35°C in the dark (Frenkel et al., 2016; Lukowska-Chojnacka et al., 2016; Li et al., 2017).

For survival assay, four groups of larvae were pretreated as described above. Survival was recorded every day for 4 days. Larva was considered dead if they gave no response to slight touch with forceps.

For fungal burden determination, another four groups of larvae were pretreated as described above. Three larvae were randomly taken from per group daily over 4 days and then homogenized in 3 ml sterile PBS/penicillin sodium using a homogenizer. Subsequently, fungal burden of each group was determined by colony counting methods (Krezdorn et al., 2014).

For histological study, four groups of larvae were pretreated as described above and a group of larvae was treated as the blank group without injectant. After 2 days of incubation, two larvae were taken randomly from each group and then were immersed in 4% paraformaldehyde fixative overnight. Subsequently, larvae were fixed in tissue OCT-freeze medium and cut into 14 μm tissue sections using a freezing microtome (Gu et al., 2016). The tissue sections then were stained with Periodic acid Schiff (PAS) and were observed under a microscope.

### Hyphal Growth Assay

Hyphal growth assay was performed in hypha-inducing media, RPMI1640 and spider medium in the well-plate (Li et al., 2014a; Haque et al., 2016). *C. albicans* suspension (2 × 10^5^ CFU/ml) was treated with different concentrations of NBP (32, 64, and 128 μg/ml) at 35°C for 4 h. The group treated without NBP was served as a control group. The cell suspension was then aspirated and each well was washed with 200 μl PBS to remove the nonadherent cells. The samples were examined under bright field using 20X objective lens by TH4-200 fluorescence microscope (Olympus, Japan) and photographed.

### Measurement of Reactive Oxygen Species Levels Assay

The levels of ROS produced by *C. albicans* treated with different concentrations of NBP were measured using the DCFH-DA (MedChem Express, USA). *C. albicans* suspension (5 × 10^5^ CFU/ml) was treated with NBP (32, 64, and 128 μg/ml) for 4 h and the group treated without NBP was set as a control group. The cells were then washed with PBS, incubated with 40 μM DCFH-DA in the dark for 30 min and detected by a BD FACS Aria II flow cytometer (Becton Dickinson, USA) with an excitation wavelength at 488 nm and emission wavelength at 530 nm.

### Analysis of Δ*ψ_m_*

Rhodamine123 (Rh123, Sigma, USA) was used to examine the effect of NBP on the *C. albicans* Δ*ψ_m_* in this study (Zheng et al., 2018). The yeast cells were pretreated with different concentrations of NBP as described in “Measurement of ROS levels assay.” The cells were stained then with 15 μM Rh123 for 30 min in the dark and detected by the flow cytometer with an excitation wavelength at 488 nm and emission wavelength at 530 nm.

### Rh6G Uptake and Efflux Assay

The drug uptake and efflux of *C. albicans* were measured by Rh6G assay due to both Rh6G and FLC are substrates of drug transporters (Pina-Vaz et al., 2005). *C. albicans* suspension (10^7^CFU/ml) was first de-energized for 1 h in PBS (without glucose), collected, and resuspended again to obtain the concentration as above.

For Rh6G uptake assay, final concentrations of 10 μM Rh6G and 32 μg/ml NBP were added to the de-energized cells simultaneously, and cells without NBP were served as the control group. The mean fluorescence intensity (MFI) of intracellular Rh6G was measured every 10 min for a total of 60 min by the flow cytometer with excitation wavelength at 488 nm and emission wavelength at 530 nm.

For Rh6G efflux assay, Rh6G was added to the de-energized cells suspension at a final concentration of 10 mM. The samples were incubated in a shaking incubator at 35°C for 1 h and later transferred to an ice-water bath for 30 min to stop the uptake of Rh6G. Then cells were collected, washed and resuspended in glucose/PBS (5%). At the same time, NBP at a final concentration of 32 μg/ml was added and Rh6G alone served as the control group. At special time intervals (0.40, 80, 120, 160, and 200 min), the MFI of intracellular Rh6G was measured using a flow cytometer with excitation wavelength at 488 nm and emission wavelength at 530 nm (Peralta et al., 2012).

### Real-Time Quantitative Polymerase Chain Reaction

*C. albicans* suspension (5 × 10^5^ CFU/ml) was treated with 32 μg/ml NBP diluted with sabouraud liquid medium for 16–18 h and the group treated without NBP was set as a control group. *C. albicans* cells were collected, washed, and total RNA was isolated by the E.Z.N.A Yeast RNA kit (e9080, OMEGA). Diluted RNA was then treated with PrimeScript RT reagent kit (RR047A, TaKaRa Biotechnology) to obtain cDNA through a reverse transcription reaction. The thermal cycling condition was 95°C for 30 s as an initial denaturation step, followed by 40 cycles of 95°C for 10 s and 60°C for 34 s and ended by the melting conditions of 95°C for 15 s, 60°C for 1 min and 95°C for 15 s. The expression of each gene was normalized to that of the *ACT1* gene. Drug transporter encoding genes, *CDR1, CDR2*, and *MDR1* were determined by the RT-PCR assay as mentioned above. Sequences of the primers are listed in Table 1. The result was calculated using the 2^−(ΔΔCt)^ method (Pfaffl, 2001).

**Table 1:** Primers used in this study.

Genes	Primer sequences (5′→3′)
ACT1	F: TGGACGGTGAAGAAGTTGCT
	R: TTGGATTGGGCTTCATCACCA
CDR1	F: CCATGACTCCTGCTACCGTG
	R: CCATCGAGACCAACCCAACA
CDR2	F: TGCTGAACCGACAGACTCAG
	R: GACCAGCCAATACCCCACAA
MDR1	F: AGTTGCTTGGGGTAGTTCCG
	R: TGCTCTCAACTTTGGTCCGT

### Statistics

All experiments were performed at least three times independently. Graphs and statistical analyses were performed with GraphPad Prism 7 (GraphPad, La Jolla, CA) and IBM SPSS Statistics 22 (SPSS, Chicago, IL). All the experimental data measuring by the flow cytometer were analyzed by BD FACSDiva v6.1.3 and FlowJo v7.10.1 software. Fungal burden, rhodamine 6G uptake and efflux and relative expression levels of genes was analyzed using an unpaired *t*-test. The levels of ROS and Δ*ψ_m_* were analyzed using one-way analysis of variance (ANOVA). *p* < 0.05 was considered significant.

## Results

### Minimum Inhibitory Concentrations of N-Butylphthalide Alone and in Combination With Fluconazole Against *C. albicans*

The MICs of NBP and FLC, alone and in combination against the six tested *C. albicans* isolates, were listed in Table 2. NBP exhibited antifungal activity against *C. albicans* with MICs of 128 μg/ml, and also exhibited synergistic effects combined with FLC against resistant *C. albicans* with FICIs of 0.25, resulting in a decrease in the MICs of NBP from 128 to 32 μg/ml and the MICs of FLC from >512 to 0.25–1 μg/ml. Besides, although no synergism was observed with FICIs of >0.5 when NBP combined with FLC against susceptible *C. albicans*, the MICs of NBP could decrease from 128 to 8–64 μg/ml and the MICs of FLC could decrease from 0.5–1 to 0.25–0.5 μg/ml.

**Table 2:** Drugs interactions of NBP and FLC against *C. albicans in vitro*.

Strain^a^	MIC_80_(μg/ml)^b^				FICI^b^	Interpretation^c^
	Alone		Combined			
	NBP	FLC	NBP_comb_	FLC_comb_		
CA4	128	0.5	8	0.25	0.56	NI
CA8	128	1	64	0.5	1	NI
CA10	128	>512	32	0.25	0.25	SYN
CA16	128	>512	32	1	0.25	SYN
CA103	128	>512	32	0.25	0.25	SYN
CA632	128	>512	32	0.25	0.25	SYN

a *CA, Candida albicans*.

b *NBP, n-butylphthalide; FLC, fluconazole; FICI, fractional inhibitory concentration index*.

c *SYN, synergism; NI, no interaction*.

*MIC_80_ denotes minimum inhibitory concentration of drug that inhibited fungal growth by 80% compared with the growth control*.

*MIC_80_ values and FICIs are shown as the median of three independent experiments*.

### Sessile Minimum Inhibitory Concentrations of N-Butylphthalide Alone and Combined With Fluconazole Against *C. albicans* Biofilms

The sMICs of NBP and FLC, alone and in combination against the biofilms formed by *C. albicans* isolates, CA4, 10, and 16, were listed in Table 3. NBP exhibited anti-biofilm activity against *C. albicans* biofilms pre-formed <12 h with sMICs of 128-256 μg/ml, and also exhibited synergistic effects combined with FLC against resistant *C. albicans* biofilms pre-formed <12 h with FICIs <0.5, resulting in a decrease in the sMICs of NBP from 128–256 to 32–64 μg/ml and the sMICs of FLC from >1,024 to 0.5–8 μg/ml. Furthermore, although no synergism was observed when NBP combined with FLC against susceptible *C. albicans* biofilms pre-formed <12 h with FICIs of >0.5, the sMICs of NBP could decrease from 128 to 64 μg/ml and the sMICs of FLC could decrease from >1,024 to 0.5–4 μg/ml. In addition, NBP alone or combined with FLC hardly inhibited mature biofilms pre-formed over more than 24 h, demonstrating the limitation of NBP; being that it only has antifungal activity against immature biofilms.

**Table 3:** Drugs interactions of NBP and FLC against preformed *C. albicans* biofilms *in vitro*.

Strain^a^	Time(h)^b^	sMIC_80_(μg/ml)^c^				FICI^c^	Interpretation^d^
		Alone	Combined		
		NBP	FLC	NBP	FLC		
CA4	4	128	>1,024	64	0.5	>0.5	NI
	8	128	>1,024	64	0.5	>0.5	NI
	12	128	>1,024	64	4	>0.5	NI
	24	>512	>1,024	>512	>256	1.3	NI
CA10	4	128	>1,024	32	0.5	0.25	SYN
	8	128	>1,024	32	4	0.26	SYN
	12	256	>1,024	64	8	0.27	SYN
	24	>512	>1,024	>512	>1,024	2	NI
CA16	4	128	>1,024	32	0.5	0.25	SYN
	8	128	>1,024	32	2	0.25	SYN
	12	256	>1,024	64	4	0.26	SYN
	24	>512	>1,024	>512	>1,024	2	NI

a *CA, Candida albicans*.

b *Time indicates incubation period of preformed biofilms*.

c *NBP, n-butylphthalide; FLC, fluconazole; FICI, fractional inhibitory concentration index*.

d *SYN, synergism; NI, no interaction*.

*sMIC_80_ denotes sessile minimum inhibitory concentration of drug that produced an 80% reduction of biofilms metabolic activity compared with the growth control. sMIC_80_ values and FICIs are shown as the median of three independent experiments*.

### Time-Killing Curves

**Figure 2:**
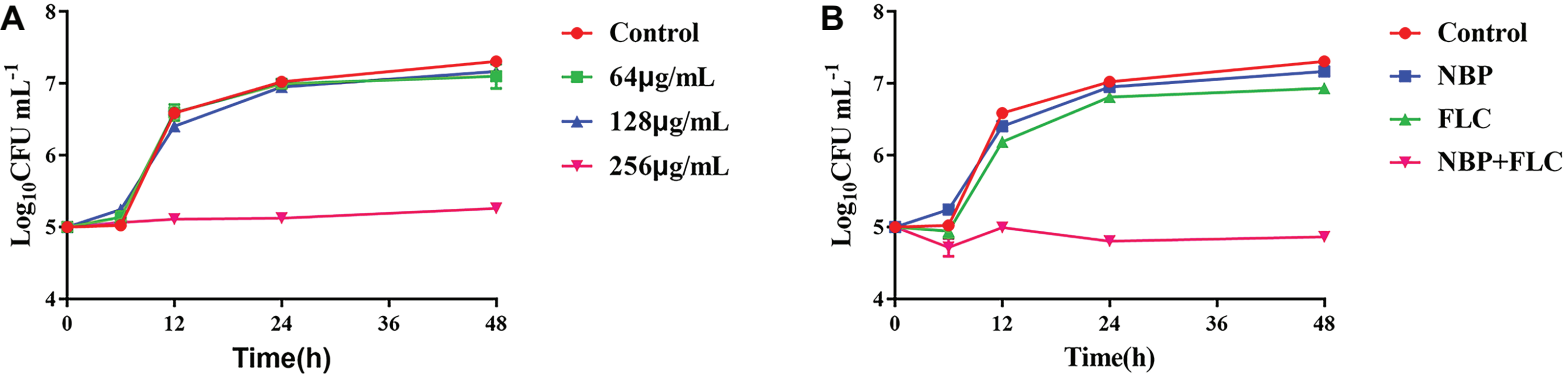
Time-killing curves of NBP alone **(A)** and in combination with FLC **(B)** against *C. albicans*. The initial yeast concentrations were adjusted to 10^5^CFU/mL. **(A)** The concentrations of NBP were 64, 128, and 256 μg/ml, respectively. **(B)** The concentrations of NBP were 128 μg/ml when combined with FLC (1 μg/ml). Values represent the means ± standard deviation of three replicates.

The results showed that, in the presence of 256 μg/ml NBP, a significant enhancement in the degree of antifungal activity was observed after 6 h, and there was a 1.91/2.05 log_10_ CFU ml^−1^ decrease at 24/48 h time point compared with the control group (Figure 2A). For the drug combination experiment, a fungal growth delay could be seen in the FLC alone group, however, it was more evident in the combination group and there was a 2.01/2.07 log_10_ CFU ml^−1^ decrease at 24/48 h time point compared with the FLC alone group (Figure 2B), indicating a synergistic antifungal effect in dynamic.

### Antifungal Effects of N-Butylphthalide Against *C. albicans in vivo*

**Figure 3:**
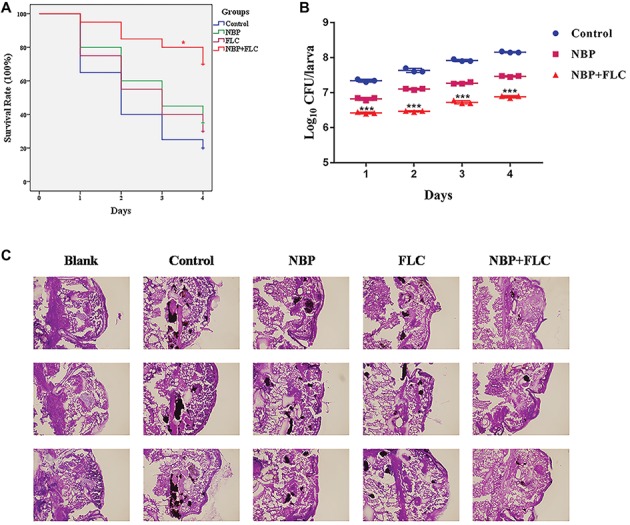
Effect of NBP in combination with FLC during *G. mellonella* infection with *C. albicans.* Larvae were injected with the inoculum of a cell concentration of 10^6^CFU/larva and then treated with PBS (control), FLC (160 μg/ml), NBP (40 μg/ml), or FLC (160 μg/ml) combined with NBP (40 μg/ml). **(A)** Survival curve of *G. mellonella i*nfected with *C. albicans*. Values represent the means ± standard deviation of three replicates. ^*^p < 0.05 compared with the NBP-treated group (Kaplan-Meier test). **(B)** Fungal burdern of *G. mellonella* infected with *C. albicans*. Data for the treatment of FLC monotherapy group were not showed because the data were similar with that of the NBP group. Values represent the means ± standard deviation of three replicates. ^***^p < 0.001 compared with the NBP-treated group (unpaired *t*-test). **(C)** Histopathology of *G. mellonella* infected with *C. albicans*. Larvae of the blank group were treated with neither *C. albicans* nor drugs. Melanized nodules containing yeast clusters and filaments could be observed except in blank group. The photographs were collected from three independent experiments.

The survival rate is the most important index to evaluate the effect of drugs *in vivo* with *G. mellonella* infection model. After 4 days of incubation, the control group showed a survival rate of 20%, while NBP group showed a survival rate of 35%, which was higher than the control group. The combinations of NBP and FLC significantly enhanced the survival rate to 70% compared with the NBP group (*p* < 0.05) (Figure 3A).

The fungal burden analysis suggested that the larval fungal burden increased gradually over 4 days after injection in all groups. Treatment with NBP slightly decreased fungal burden than the control group and data of FLC monotherapy group were not shown in figure because the data were similar with those of the NBP monotherapy group. The combination of NBP and FLC significantly reduced fungal burden compared with the NBP group (*p*’s of all 4 days was <0.001), especially in the last 3 days (Figure 3B).

Histopathology studies (Figure 3C) revealed that in comparison with the blank group, the *C. albicans* cells mainly existed in the form of filamentous clusters in larval tissues after infection. In the drug monotherapy groups, although the amounts of melanized nodules were similar with the control group, the size of these melanized nodules of these two groups was smaller than those of the control group. However, only few small melanized nodules were discovered in the drug combination group.

### Effects of N-Butylphthalide on *C. albicans* Hyphal Growth

**Figure 4:**
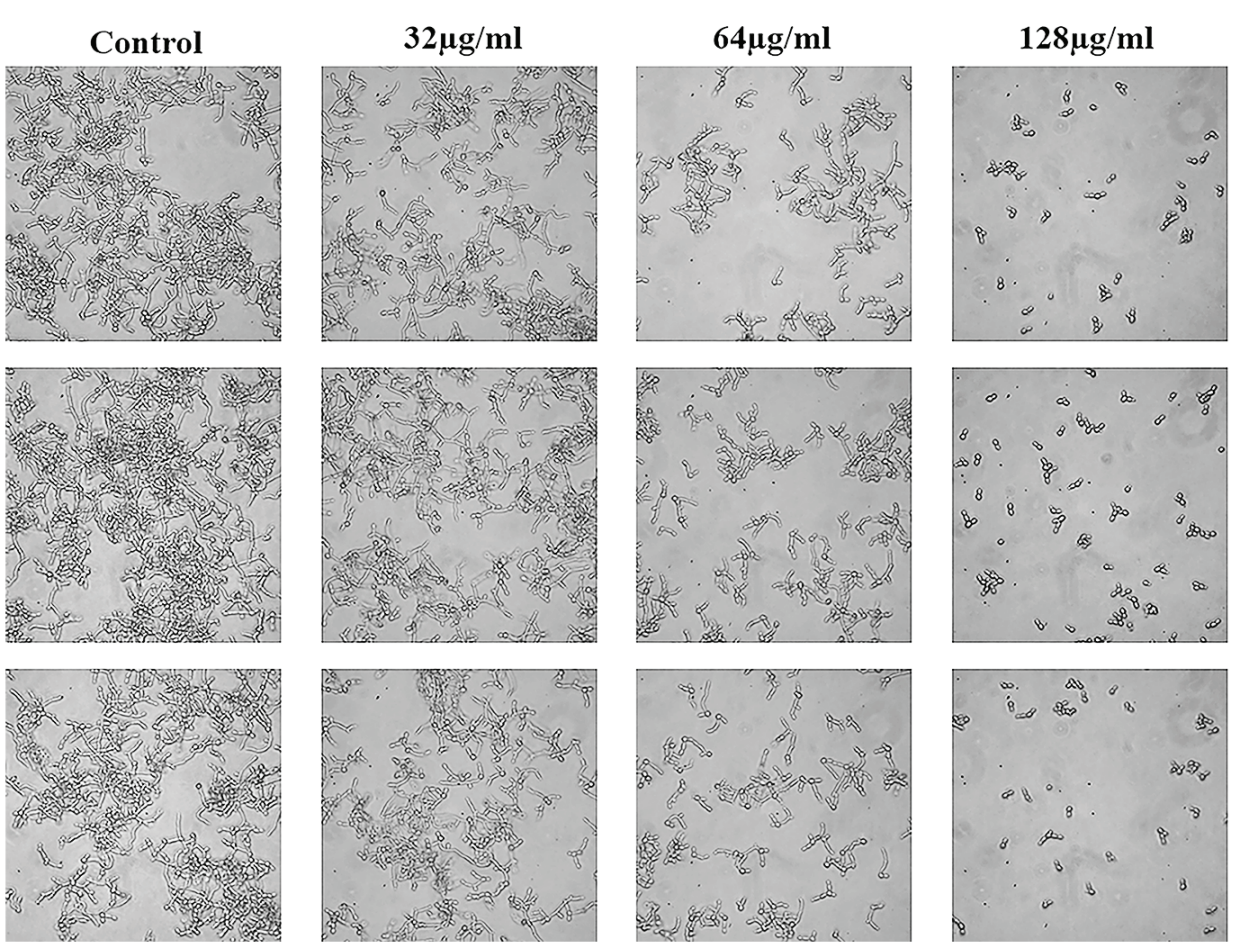
Effect of NBP on *C. albicans* hyphal growth induced by RPMI 1640 medium. NBP was diluted in hyphae-inducing media, RPMI 1640 medium, at a final concentration of 0, 32, 64, and 128 μg/ml, respectively. The cellular morphology was photographed after incubation at 37°C for 4 h. The photographs were collected from three independent experiments.

**Figure 5:**
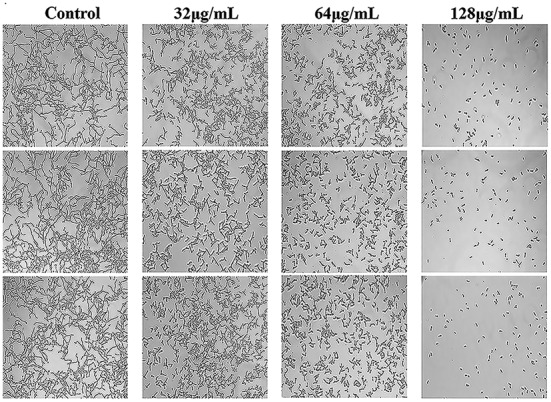
Effect of NBP on *C. albicans* hyphal growth induced by spider medium. NBP was diluted in hyphae-inducing media, spider medium, at a final concentration of 0, 32, 64, and 128 μg/ml, respectively. The cellular morphology was photographed after incubation at 37°C for 4 h. The photographs were collected from three independent experiments.

*C. albicans* hyphae induced by RPMI 1640 medium and spider medium were shown in Figures 4, 5, respectively. As shown, *C. albicans* could form long and interlaced hyphae in both RPMI 1640 and spider medium, and NBP inhibited *C. albicans* hyphal growth in both media tested in a dose dependent manner. 32 μg/ml NBP could lead to form slightly shorter hyphae than the control group. 64 μg/ml NBP could induce to form loose and patchy hyphae, while when the concentration increased to 128 μg/ml, cells were mainly maintained as yeasts and few filament could be observed in the field of vision.

### Effects of N-Butylphthalide on Reactive Oxygen Species Production

**Figure 6:**
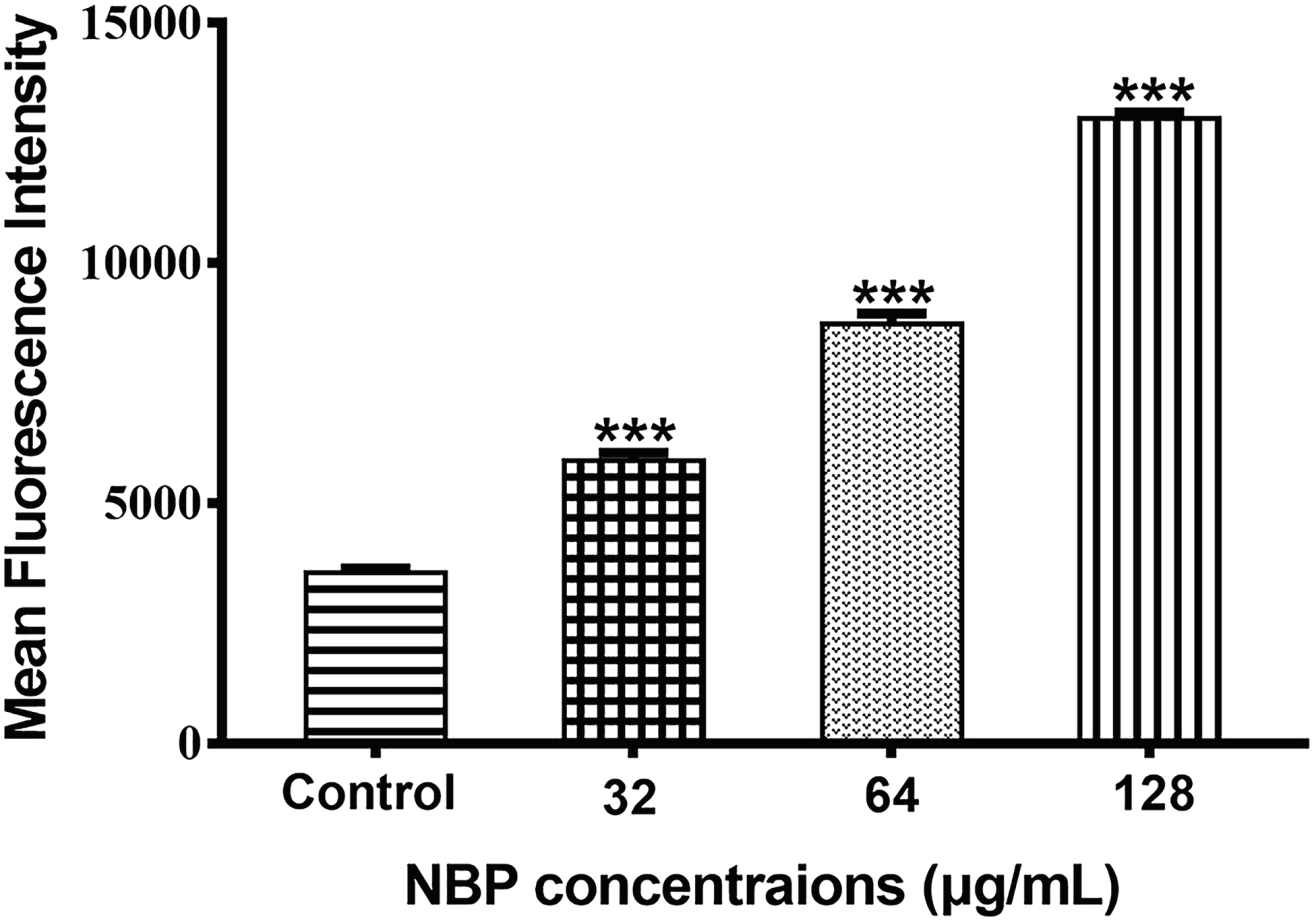
Effect of NBP on the levels of ROS in *C. albicans*. Yeast cells pretreated with diverse concentrations of NBP (0, 32, 64, and 128 μg/ml) for 4 h were incubated with 40 μM DCFH-DA and then analyzed by a flow cytometer. Values represent the means ± standard deviation of three replicates.^***^p < 0.001 compared with the control group (one-way ANOVA).

The generation of excessive ROS is considered as a potential fungicidal mechanism and we measured the levels of intracellular ROS after treated with NBP. The results showed that NBP significantly induced intracellular ROS accumulation of *C. albicans* in a dose-dependent manner (*p* < 0.001) (Figure 6). The levels of ROS resulted in approximately 165, 245, and 283% increases in MFI after treated with 32, 64, and 128 μg/ml NBP, respectively, compared with that of control group.

### Effects of N-Butylphthalide on Δ*ψ_m_*

**Figure 7:**
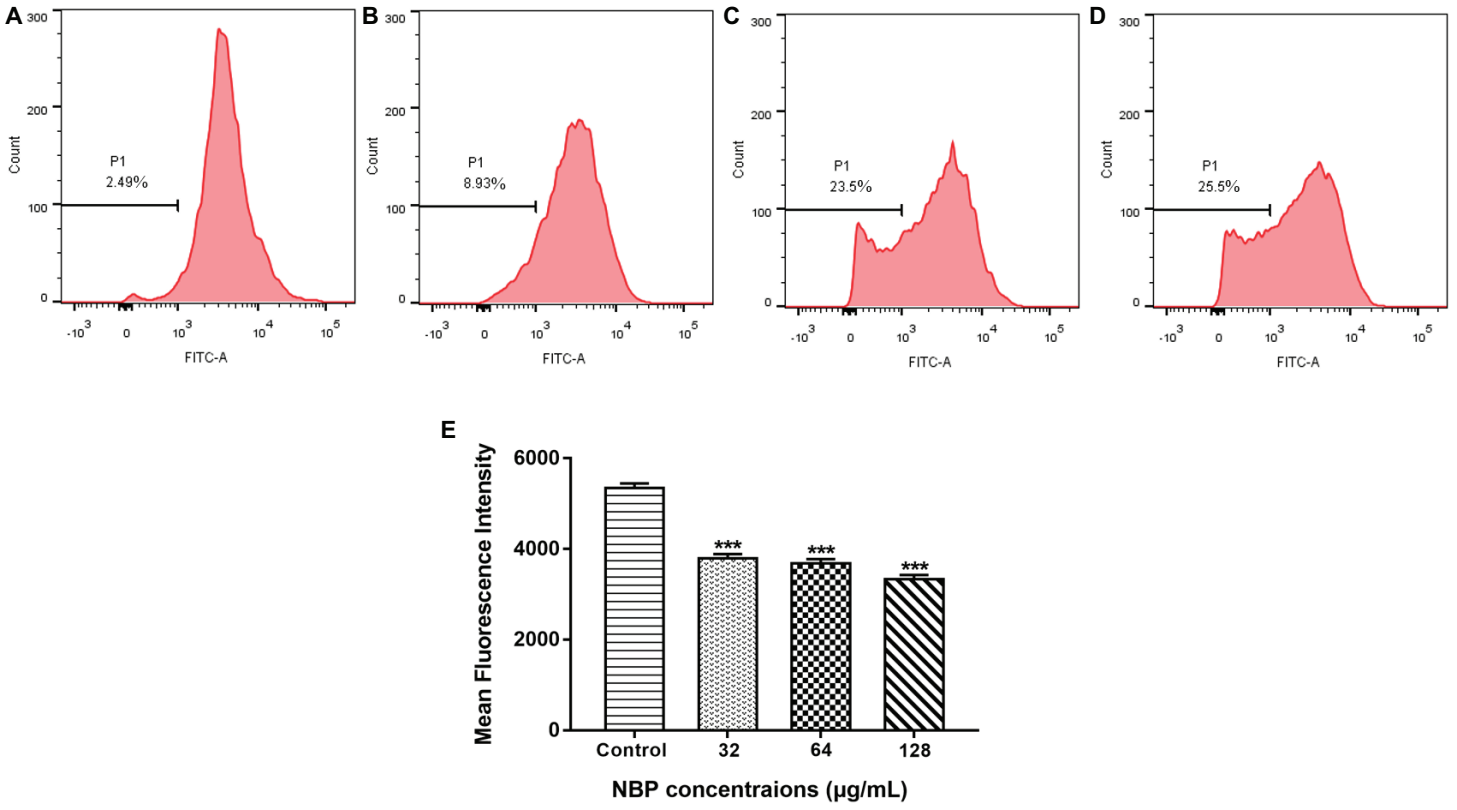
Effect of NBP on the Δ*ψ_m_* of *C. albicans.* Yeast cells pretreated with diverse concentrations of NBP (0, 32, 64, and 128 μg/ml) for 4 h were incubated with 15 μM Rh123 and then analyzed by a flow cytometer. **(A–D)** Effect of different doses of NBP on Δ*ψ_m_* loss in *C. albicans.* **(A)** Negative control, **(B)** 64, **(C)** 128, **(D)** 256 μg/ml. Representative images from three independent experiments are shown. The percentages of cells in the left section of the histogram refer to the number of cells with depolarized mitochondria. **(E)** Effect of NBP on the mean fluorescence intensity of Rh123-stained *C. albicans*. ^***^p < 0.001 compared with the control group (one-way ANOVA).

To determine whether the NBP induced ROS accumulation was involved in the metabolic state of mitochondria, Δ*ψ_m_* treated with NBP was measured. As shown in Figures 7A–D, NBP significantly reduced the number of *C. albicans* cells with an intact Δ*ψ_m_* and increased the number of cells with low Δ*ψ_m_* in a dose dependent manner. The percentage of *C. albicans* with reduced Δ*ψ_m_* increased from 2.49% in the control group to 8.93, 23.5, and 25.5% at 32, 64, and 128 μg/ml NBP, respectively. Besides, NBP obviously reduced intracellular Rh123 MFI of *C. albicans* in a dose dependent manner (Figure 7E), also suggesting a decrease in Δ*ψ_m_*. Thus, NBP induced a loss in Δ*ψ_m_* of *C. albicans* and consequently caused mitochondrial dysfunction.

### Effects of N-Butylphthalide on Drug Uptake and Efflux of *C. albicans* and Drug Transporters Genes

**Figure 8:**
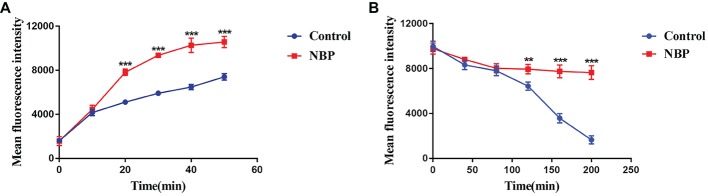
Effect of NBP on drug uptake **(A)** and efflux **(B)** of *C. albicans* tested by Rh6G assay. The concentration of NBP is 32 μg/ml and cells treated without NBP were served as the control group. Mean fluorescence intensity represented intracellular Rh6G and were measured by a flow cytometer. Values represent the means ± standard deviation of three replicates. ^**^p < 0.01 and ^***^p < 0.001 compared with the control group (unpaired *t*-test).

**Figure 9:**
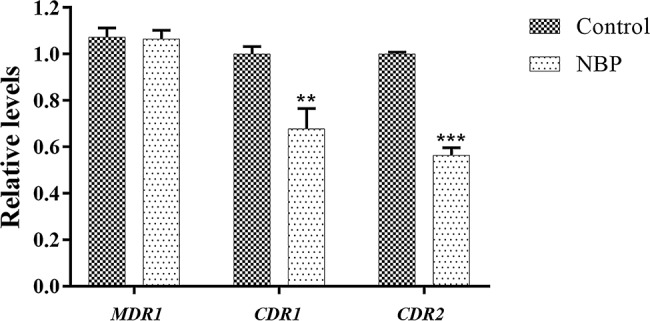
Relative expressions of drug transporter encoding genes *MDR1, CDR1*, and *CDR2*. The concentration of NBP is 32 μg/l and cells treated without NBP were served as the control group. Values represent the means ± standard deviation of three replicates. ^**^p < 0.01 and ^***^p < 0.001 compared with the respective control group (unpaired *t*-test).

In the drug uptake experiment, NBP significantly increased drug absorption after 20 min and cells in the NBP-treated group absorbed higher concentrations of Rh6G than the control group (*p* < 0.001) (Figure 8A). In the drug efflux experiment, after treated with glucose solution, the MFI both in the control group and NBP-treated group showed a downward trend. However, NBP evidently suppressed the decrease especially after 120 min (*p* < 0.001) and cells treated with NBP pumped out much lower concentration of Rh6G compared with the control group in 200 min (Figure 8B). Subsequently, gene expression experiments confirmed that NBP significantly down-regulated the expression levels of genes, *CDR1* and *CDR2*, that encode *C. albicans* drug resistant protein (Cdrp), however, for the expression level of *MDR1*, there was no difference between the NBP-treated group and the control group (Figure 9). In a word, the results of Rh6G assay and RT-PCR method suggest that the synergistic antifungal effect of NBP and FLC was related to promote drug uptake and reverse drug efflux *via* down-regulating drug transporters genes, *CDR1* and *CDR2*.

## Discussion

Treatment of fungal infections, especially candidiasis, is still a challenging problem due to the rising isolation rates of resistant strains (Pfaller and Diekema, 2007). In our previous studies, we focused on studying the mechanism of drug resistance in *C. albicans* and exploring more non-antifungal drugs with antifungal activity to overcome drug resistance of *C. albicans*, especially sensitizers of traditional antifungal drugs, such as antibiotics, glucocorticoid and calcium channel blockers (Liu et al., 2016; Sun et al., 2017; Lu et al., 2018). In this study, we demonstrated NBP, a component of *Apium graveolens* seeds and currently used for the treatment of ischemic stroke in clinic, exerted antifungal activity against *C. albicans* with MICs of 128 μg/ml and NBP combined with FLC showed synergistic effects against resistant *C. albicans*, leading to a decrease in the MICs of FLC from >512 to 0.25–1 μg/ml. Besides, time-killing curves provided figures that described dynamic antifungal effects of NBP alone and synergistically combined with FLC on *C. albicans*, which was in accordance with those from broth microdilution assays.

Biofilms, consisting of complicated communities of microorganisms embedded in cells-derived matrix, build a heterogeneous and natural drug-tolerant environment (Bonhomme and d’Enfert, 2013). *C. albicans* biofilms, especially formed on medical implants, are common during *C. albicans* infections and pose a great challenge to clinical treatment due to the intrinsic resistance to most antifungal drugs (Mathe and Van Dijck, 2013). We demonstrated that NBP exerted anti-biofilm activity against *C. albicans* biofilms pre-formed <12 h with sMICs of 128–256 μg/ml and NBP combined with FLC showed synergistic effects against resistant *C. albicans* biofilms pre-formed <12 h, leading to a decrease in the sMICs of FLC from >1,024 to 0.5–8 μg/ml. These results indicated that NBP has broad prospects in prevention and treatment of immature *C. albicans* biofilms related infections.

The *G. mellonella* model, whose immune response is similar with that of mammals, is used as an infection model host and *Galleria mellonella-Candida albicans* infection model is widely applicable to rapidly evaluate the efficacy of drugs against *C. albicans* infections *in vivo* (Li et al., 2013; Aneja et al., 2016). Compared with mammalian model, *G. mellonella* model has many advantages, such as significant ethical, economical, accessible and easy manipulative (Mylonakis et al., 2007; Vilcinskas, 2011). Besides, the *G. mellonella* larvae infection model belongs to invertebrate and does not require ethical approval. In the present study, we found that NBP monotherapy enhanced survival rate of *C. albicans* infected larvae, cleared more *C. albicans* cells in larvae and caused less damage to larval tissues compared with the control group, while the combined treatment of NBP and FLC exhibited a better therapeutic effects. Acute toxicity test indicated that median lethal dose of NBP to mice was much higher than the effective dose; moreover, chronic toxicity test excluded chronic adverse events during long-term application of NBP (Tian et al., 2016). The doses (0.4 μg/larva for NBP and 1.6 μg/larva for FLC) used to treat larvae was converted from those used to treated human beings. Thus, in consideration of therapeutic effects on *G. mellonella* infection model and good safety of NBP, it is promising for application of NBP combined with antifungal drugs against *C. albicans* infections, while more researches are needed to be carried out in future.

For the potential mechanisms exploration, we first measured the effects of NBP on hyphal growth. As a dimorphic fungus, *C. albicans* proliferated in either a yeast form or a hypha form (Wilson et al., 2016). Hyphae are crucial components of *C. albicans* biofilms and required for virulence and pathogenicity, contributing to adhesion and invasion of host cells (Whiteway and Bachewich, 2007). The results showed that the morphological characteristics of *C. albicans* hyphae, induced by both RPMI 1640 and spider medium, were similar and NBP inhibited *C. albicans* hyphal growth in a dose-dependent manner. Hyphae induced by 64 μg/ml NBP were shorter and looser than that of control group, while 128 μg/ml NBP obviously inhibited the yeast-to-hypha morphological transition and *C. albicans* were mainly maintained in yeast form. Thus, NBP exhibited antifungal activity *via* inhibiting hyphal growth, attenuating virulence factors and reducing invasiveness and pathogenicity.

Generation of ROS regulates the process of apoptosis in eukaryotes, leading to enzyme inactivation, cells dysfunction and subsequent cell death (Perrone et al., 2008; Chen et al., 2013). Mitochondria is a major subcellular source of intracellular ROS. Δ*Ψ_m_* plays a coupling role in mitochondrial oxidative phosphorylation and its stability is beneficial to maintain regular physiological functions of cells (Dai et al., 2015; Zhu et al., 2015). In this study, NBP significantly induced *C. albicans* intracellular ROS accumulation and a loss in *C. albicans* Δ*ψ_m_* in a dose-dependent manner. Recent studies showed the induction of excessive ROS in *C. albicans* was a critical factor in cell death induced by many antifungal drugs (Chen et al., 2013; Chang et al., 2017). Depolarization of the mitochondrial membrane indicates the change of Δ*ψ_m_* and the subsequent flow of the outer membrane is also a critical stage in the intrinsic apoptosis pathway (Chen et al., 2015; Wang et al., 2015). Thus, the results suggested NBP induced *C. albicans* cells death was probably triggered by stimulated intracellular ROS accumulation and dysfunction of mitochondrion, finally leading to cell death through mediation of apoptosis.

It is extensively accepted that resistance of *C. albicans* to most antifungal drugs is partly mediated by the efflux mechanism. The non-specificity of drug efflux pump transporters possibly explains the phenomenon of cross-resistance between diverse antifungal drugs (Pina-Vaz et al., 2005). Generous studies demonstrated that inhibiting the activity of drug efflux pumps might be a non-negligible mechanism to illustrate the synergism of interactions between non-antifungal drugs and conventional antifungal drugs (Pina-Vaz et al., 2005; Li et al., 2017). In this study, the results suggested that NBP significantly promoted drug uptake of *C. albicans.* Indeed, *C. albicans* could agglomerate and adhere to form biofilms that are important biological barrier for drug diffusion and inherent resistant to most antifungal therapy. With the formation of biofilms, cell viscosity increases, and drug diffusion slows down. NBP could inhibit the *C. albicans* biofilms, which may be one of the reasons why NBP promotes drug uptake by destroying the physical barrier of biofilms. Subsequently, it was demonstrated that NBP suppressed drug efflux, led to high levels of FLC concentrations in fungal cells and then possibly increased the susceptibility of *C. albicans* to FLC. RT-PCR method further confirmed that NBP down-regulated the expressions of drug transporters genes *CDR1* and *CDR2*. Thus, the synergistic effect of NBP and FLC relied on promoting drug uptake and reversing the mechanism of drug efflux targeting drug transporters genes *CDR1* and *CDR2*.

In summary, NBP alone exhibited antifungal activity against both planktonic *C. albicans* and biofilms. Strong synergism was observed when NBP combined with FLC against resistant *C. albicans* and biofilms pre-formed by resistant strains. Time-killing curves confirmed antifungal effects of NBP in dynamic. The antifungal activity of NBP was further confirmed *in vivo* with *G. mellonella* infection model. Mechanism researches showed that NBP could inhibit the hyphal growth, induced intracellular ROS accumulation and caused mitochondrial dysfunction. Besides, the mechanism of synergism between NBP and FLC relied on promotion of drug uptake, suppression drug efflux by down-regulating drug transporters genes *CDR1* and *CDR2*. To the best of our knowledge, this study is the first to elucidate the antifungal activity of NBP both *in vitro* and *in vivo* and explore the potential mechanisms. These findings might provide insights into the possible therapeutic application of NBP as antifungal agents or sensitizers of traditional antifungal drugs in the future.

## Author Contributions

YG, WL, and SS designed the experiments. YG and YL performed the experiments. YG, XH, and LH interpreted the data. YG and SS wrote the manuscript. All authors approved the manuscript for publication.

### Conflict of Interest Statement

The authors declare that the research was conducted in the absence of any commercial or financial relationships that could be construed as a potential conflict of interest.
